# ERN GENTURIS guideline on counselling on reproductive options for individuals with a cancer predisposition syndrome (including genturis)

**DOI:** 10.1038/s41431-025-02007-4

**Published:** 2026-01-13

**Authors:** Said C. Farschtschi, Candy Kumps, Tamara Hussong Milagre, Periklis Makrythanasis, Ariane Van Tongerloo, Ellen Denayer, Mariëtte van Kouwen, Estela Carrasco López, Anna Sophie Berghoff, Salvo Testa, Claudia Cesaretti, Eva Trevisson, Renata d’ Oliveira, Francesca Fianchi, Claas Röhl, Diana Salinas-Chaparro, Ileen Slegers, Marianne Geilswijk, Manon Suerink, Irene Spinelli, Sandra Janssens, Sarah Pugh, Laura Kirstine Sønderberg Roos

**Affiliations:** 1 University Medical Center Hamburg-Eppendorf, Hamburg, Germany; 2 University Hospital Ghent, Ghent, Belgium; 3 EVITA Association – Hereditary Cancer (Associação EVITA – Cancro Hereditário, Lisbon, Portugal; 4 Laboratory of Medical Genetics, ‘Aghia Sophia’ Children’s Hospital, National and Kapodistrian University of Athens, Athens, Greece; 5 Department of Genetic Medicine and Development, Medical School, University of Geneva, Geneva, Switzerland; 6 Biomedical Research Foundation of the Academy of Athens, Athens, Greece; 7 Center for Human Genetics, University Hospitals Leuven, University of Leuven, Leuven, Belgium; 8 Radboud university medical center, Nijmegen, the Netherlands; 9 Vall d’Hebron University Hospital, Barcelona, Spain; 10 Medical University of Vienna, Vienna, Austria; 11 Fondazione Mutagens (hereditary syndromes carriers), Milano, Italy; 12 Fondazione IRCCS Ca’ Granda, Ospedale Maggiore Policlinico, Milan, Italy; 13 Department of Women’s and Children’s Health, University of Padua, University Hospital of Padua, Padua, Italy; 14 Unidade Local de Saúde (ULS) São João, Porto, Portugal; 15 Fondazione Policlinico Universitario A. Gemelli IRCCS, Rome, Italy; 16 NF Kinder/ NF Patients United, Vienna, Austria; 17 Hospital Sant Joan de Deu, Barcelona, Spain; 18 UZ Brussel, Brussel, Belgium; 19 Aarhus University Hospital, Aarhus, Denmark; 20 Leiden University Medical Center, Leiden, the Netherlands; 21 Manchester Centre for Genomic Medicine, Manchester University Foundation NHS trust, Manchester, UK; 22 Rigshospitalet, Copenhagen, Denmark

**Keywords:** Genetic counselling, Health policy

## Abstract

Cancer predisposition syndromes (CPSs), including genetic tumour risk syndromes (genturis), are a heterogeneous group of genetic disorders characterised by an increased risk of developing tumours compared to the general population. CPSs raise reproductive issues for affected individuals because of the risk of passing the disease-causing genetic alterations on to offspring. The demand for reproductive counselling is often unmet due to the lack of sufficient healthcare professionals with the specialised knowledge, experience and skill. Based on a comprehensive literature review of 851 publications and expert consensus (multidisciplinary medical experts and patient representatives), the European Reference Network on genetic tumour risk syndromes (ERN GENTURIS) developed a guideline providing 16 recommendations for reproductive counselling in CPSs. The central recommendation is to offer reproductive counselling proactively to all individuals with a CPS and their relevant family members, together with psychological support and in multidisciplinary collaborations. This guideline aims to standardize the offer of reproductive counselling for individuals with a CPS across Europe, empowers healthcare professionals for their specific tasks, and helps patients dealing with their own challenges.

## Introduction

Cancer predisposition syndromes (CPSs) (including genetic tumour risk syndromes (genturis; CPSs included in the European Reference Network on Genetic Tumour Risk Syndromes (ERN GENTURIS [1]))), e.g., hereditary breast and ovarian cancer, Li-Fraumeni syndrome, neurofibromatosis type 1, schwannomatosis and Lynch syndrome ([2, 3], www.genturis.eu/l=eng/thematic-disease-groups.html), affect up to 10% of cancer patients [4]. CPSs often present with early-onset tumours and have risk of transmission of the genetic predisposition. Individuals face dual burdens: personal cancer risks and concerns about transmitting the condition to offspring. Moreover, in syndromic conditions, non-tumorous symptoms that may influence the perception of the disease are common. For instance, neuropsychological conditions can impede genetic counselling and vice versa, genetic counselling may impact psychological issues in these conditions.

For the majority of the CPSs, the inheritance pattern is autosomal dominant, following Mendelian rules, meaning that the risk for a descendent to inherit the condition is usually 50% from each parent. Additionally, mosaicism is known for some CPSs, for which the risk of inheritance mostly is reduced (below 50%). It is important to note that while carriers of a CPS may not develop a cancer, they remain at significantly elevated risk over the course of their lifetime. The likelihood of a cancer can be influenced by variable penetrance, modifier genes, and environmental factors, underscoring that the presence of a CPS is not equivalent to certainty [4]. CPSs with a X-linked or autosomal recessive inheritance have also been reported, but they are rare, and instances arising de novo are particularly rare [5]. Individuals with a CPS who wish to have a child may have several reproductive options available to them, including prenatal diagnosis (PND), and preimplantation genetic testing for monogenic diseases (PGT-M). Some individuals with a CPS may consider not to have biological children because of the risk of passing the condition on to a child. Other options include adoption, surrogacy and gamete or embryo donation. Reproductive decision making for individuals with a CPS is inherently complex and nuanced, encompassing emotional, ethical, and practical challenges. These challenges can be further conditioned by sociocultural, legal, and health-economic contexts, which differ across countries. Despite this complex situation and the request for help from affected individuals, the availability and awareness of specialized, adequate reproductive counselling remains limited [6]. ERN GENTURIS (European Reference Network for patients with a rare and/or complex genturis) therefore developed a guideline with the aim to highlight disparities, establish standards, empower healthcare professionals and help patients in upholding their autonomy across diverse healthcare systems and social environments in Europe. Health questions addressed in this guideline are:

- What content should counselling regarding reproductive options have for individuals with a CPS?
- How can healthcare professionals aid individuals with a CPS to make informed choices?
- Under what circumstances and at what time in the person’s life should healthcare providers professionals refer individuals with a CPS for counselling regarding reproductive options?
- In what context should counselling for reproductive options be provided to individuals with a CPS?
- Who should perform counselling for reproductive options for individuals with a CPS?
- How should reproductive counselling be performed for individuals with a CPS?

## Methods

The ERN GENTURIS guideline group for counselling on reproductive options for individuals with a CPS, including genturis (ERN GENTURIS counselling on reproductive options guideline group), was established by 20 experts in reproductive counselling from 10 countries and 3 patient representatives (Supplementary Table 1). The core working group of this guideline group existed of 5 medical professionals and 1 patient representative. The core working group met online on a regular basis over 18 months and drafted the initial guideline scope, clinical questions, recommendations and a guideline document, and obtained feedback from the guideline group. The recommendations were finalised in a modified Delphi approach in which all members of the ERN GENTURIS counselling on reproductive options guideline group and 41 additional experts participated. The guideline was based on an initial review (title, abstract) on 851 publications extracted from PubMed in April/May 2024 (literature search included in supplement). Of these papers, 245 had a full paper review as they could potentially answer the health questions addressed in this guideline. In the end, 85 publications formed the basis of the 24 recommendations that entered the Delphi survey. The full details of the guideline, including literature search, reference list and Delphi process, can be found at:

https://www.genturis.eu/l=eng/Guidelines-and-pathways/Clinical-practice-guidelines.html.

The Delphi survey consisted of two rounds. Participants of this Delphi process (listed in Supplementary Table 2) assessed recommendations by a four-point Likert scale (totally disagree, disagree, agree, totally agree). Consensus was defined when >60% of participants responded “agree” or “totally agree”. However, even if consensus was met, recommendations were further modified if a higher consensus was thought to be achievable from the written responses that accompanied the ratings. All 16 recommendations included in this manuscript reached over 90% of agreement.

To balance the weight of published evidence and quantify the wealth of expert experience and knowledge, we graded the recommendations according to a three-point scale: Strong = expert consensus AND consistent evidence; Moderate = expert consensus WITH inconsistent evidence AND/OR new evidence likely to support the recommendation; Weak = Expert majority decision WITHOUT consistent evidence.

## Results

### General considerations (supplementary Table 3)

#### For counselling

Personal philosophies, religion, cultural values, and individual preferences concerning family and reproduction significantly influence attitudes towards prenatal diagnosis (PND) and preimplantation genetic testing (PGT). Counsellors should be sensitive to and understand these perspectives, ensuring non-judgemental, personalised, and non-directive support.

#### For healthcare professionals

Access to necessary information should always respect the individual’s autonomy and personal readiness. Information should be provided in a timely manner and tailored to the individual’s needs and circumstances, considering all personal and healthcare system factors. Genetic counselling should be oriented to up-to-date and evidence-based information, while also recognising that clinical screening strategies, treatment guidelines, diagnostic criteria, nomenclature, reproductive methods, and genetic techniques may change over time.

#### For healthcare centres providing counselling on reproductive options

Reproductive counselling should take place prospectively and should include realistic information on the process of all the available reproductive options, including natural conception and risk-assumption, PND, PGT, gamete donation, adoption, and postnatal predictive testing of the child. Benefits, drawbacks, limitations and challenges should be explained, such as the medical procedure, possible psychological impact, success rates, and the possibility of obtaining only affected embryos. Also, the reproductive window, waiting times and delays (due to technicalities in PGT) should be discussed. Furthermore, fertility preservation options, such as oocyte/sperm cryopreservation, can be relevant to include in reproductive counselling, especially in situations with a high risk of infertility due to cancer treatment. During counselling, realistic expectations and informed decision-making should be aimed at, tailored to the patient’s reproductive potential, before initiating any procedures. For example, a 38-year-old woman may have lower success rates in PGT-M procedures due to her ovarian reserve and oocyte quality compared to a 28-year-old woman. Liaison with IVF clinics should be provided in a timely manner. As reproductive options are regulated under strict national laws, guidance should be provided in accordance with country-specific legislations. Ethical board approval for PND or PGT may have to be obtained in advance for individuals with a CPS on an individual basis; for those cases, refusal and alternatives should be included. If applicable, information about the availability of public funding for PGT should be included.

In addition to these general considerations (Supplementary Table 3), this guideline provides recommendations in four broad and overlapping areas:

1. Reproductive decision making (Table 1) - content and framework of reproductive counselling
2. Timing of reproductive counselling provision (Table 2)
3. Presentation of reproductive options (Table 3)
4. Range of assisted reproductive technologies (Table 4).

**Table 1:** Reproductive decision making—content and framework of reproductive counselling.

Recommendations		Strength
**Rec. 1**	Reproductive counselling should be offered to all individuals with a CPS^a^. It is voluntary for the individual with a CPS to accept or decline counselling.	Strong
**Rec. 2**	All individuals with a CPS and relevant^b^ family members of reproductive age should be offered information about their reproductive options.	Strong
**Rec. 3**	Reproductive counselling must provide individuals with a CPS and relevant^b^ family members with comprehensive, balanced, and timely information.	Strong
**Rec. 4**	Reproductive counselling should be non-directive ensuring patients can freely decline specific or all reproductive options without fear of recrimination, feelings of guilt or social pressure.	Strong
**Rec. 5**	Couples, at risk for a child with a CPS, considering prenatal diagnosis^c^ should be encouraged to reflect on their views regarding continuation or termination of pregnancy preconceptionally^d^.	Moderate
**Rec. 6**	Couples with a CPS considering pregnancy should have access to a multidisciplinary team of healthcare experts in an individualised way. This may include: A genetic counsellor or clinical geneticist to assess genetic risk, discuss the feasibility of both prenatal diagnosis (PND)^c^ and IVF (in vitro fertilization) with preimplantation genetic testing (PGT)^c^. A clinician experienced in performing and interpreting prenatal diagnostic tests to explain the risks, benefits, and procedures of PND^c^ options such as amniocentesis, chorionic villus sampling, and NIPT, if PND^c^ is considered. A fertility doctor to provide guidance on PGT^c^, including PGT-M, and other assisted reproductive techniques where relevant. A psychologist trained in reproductive and genetic counselling, given the emotional and psychological impact of these decisions, In difficult or unusual cases, advice should be sought from additional experts.	Strong

^a^Counselling is especially relevant in the reproductive age but can be relevant in other age groups as well, such as adolescence and older individuals informing their relatives.

^b^Definition: relevant family members can include first-degree relatives, such as parents, children, or siblings but also second- and higher-degree relatives. It depends on the characteristics and the inheritance mode of the syndrome. The healthcare professional will determine who the relevant family members are.

^c^Definition: prenatal diagnosis refers to tests conducted to diagnose a foetus in utero, including amniocentesis, chorionic villus sampling, and NIPT (NIPT needs to be confirmed by an invasive test).

^d^Preconceptionally is before pregnancy.

**Table 2:** Timing of reproductive counselling provision.

Recommendations		Strength
**Rec. 7**	Reproductive counselling should be offered longitudinally to individuals with a CPS (and relevant^a^ family members) with multiple opportunities for discussion during reproductive age. At the time of diagnosis, individuals with a CPS should receive clear information about the availability of genetic and reproductive counselling services for future family planning.	Strong
**Rec. 8**	Genetic and reproductive counselling should be available for individuals with a CPS and for parents (at risk) of an affected child, ideally beginning before family planning and continuing as needed.	Strong
**Rec. 9**	Individuals with a CPS should be offered age- and context-appropriate genetic and reproductive counselling at the time of diagnosis.	Strong
**Rec. 10**	Children at risk for cancer susceptibility should be offered counselling^b^ regarding predictive testing and family planning once they reach adulthood, or earlier if they express interest or the condition affects childhood.	Moderate
**Rec. 11**	Counselling regarding reproductive options is relevant for all individuals with a CPS, regardless of whether they already have children, are considering more children, or are not currently planning a pregnancy, since this may influence decisions regarding testing or informing their children/family members.	Moderate

^a^Definition: relevant family members can include first-degree relatives, such as parents, children, or siblings, but also second- and higher-degree relatives. It depends on the characteristics and the inheritance mode of the syndrome. The healthcare professional will determine who the relevant family members are.

^b^Counselling in minors should be provided with parental consent and involve specialist with expertise in paediatric care. If initiated in childhood, follow-up should be continuous to adapt to the individual’s evolving understanding and needs.

**Table 3:** Presentation of reproductive options.

Recommendations		Strength
**Rec. 12**	Reproductive counselling for individuals with a CPS should provide sufficient time, follow-up opportunities, and access to psychological support.	Moderate
**Rec. 13**	Reproductive counselling should take psychological factors into account and be provided by a multidisciplinary team. This team should include professionals with expertise in reproductive genetics, oncology (when relevant), and psychological support. Access to additional specialists should be tailored to individual patient needs.	Strong
**Rec. 14**	Reproductive counselling should be offered to both male and female individuals with a CPS independently and include their partners, if appropriate.	Strong

**Table 4:** Range of assisted reproductive technologies.

Recommendations		Strength
**Rec. 15**	Female fertility preservation options, such as oocyte cryopreservation, should be included in reproductive counselling for individuals with a CPS, when there is a high risk of infertility due to cancer treatment^a^.	Strong
**Rec. 16**	Male fertility preservation options, such as sperm cryopreservation, should be included in reproductive counselling for individuals with a CPS, when there is a high risk of infertility due to cancer treatment^b^.	Moderate

^a^This discussion should be tailored to the individual’s age, ovarian reserve, and the feasibility of fertility preservation in their specific healthcare setting. Ideally, oncologists should address this topic early, before treatment begins.

^b^This discussion should ideally take place at the time of cancer diagnosis and be led by the oncology team before treatment begins. Counselling should be tailored to individual risk factors and the feasibility of fertility preservation within the specific healthcare setting.

#### Reproductive decision making—content and framework of reproductive counselling

The first set of recommendations 1–6 (Table 1) is regarding reproductive decision making, and the framework of counselling. An affected individual and the relevant family members should be informed about available tests and possible options (rec. 1, 2, 3, 14, 15, 16). Information regarding the pathogenic variant (ACMG/AMP class 5) or likely pathogenic variant (ACMG/AMP class 4), disease spectra, variability of expression, penetrance, mortality, morbidity and available surveillance programs is also important for the decision making [7–9]. Mosaicism reduces the risk of inheriting the (likely) pathogenic genetic variant and may influence the severity of symptoms; in this situation, the offspring of an asymptomatic mosaic carrier can inherit the condition and may be more severely affected [10–12].

Natural conception without genetic testing implies an acceptance of the risk of passing on the (likely) pathogenic genetic variant to offspring. In classical autosomal dominant inheritance, that risk is usually 50%. In an individual with mosaic CPS, that risk may be reduced. Choosing natural conception can also mean accepting uncertainty about the child’s genetic status until adulthood, when the child can decide whether or not to undergo predictive testing for an adult-onset condition. Current practice is that predictive testing for adult-onset conditions is generally not offered in childhood, in order to respect the child’s future autonomy. However, when a CPS is associated with childhood-onset disease or when surveillance or preventive measures are indicated during childhood, predictive testing of the child can be considered at the time point when screening or intervention is recommended (rec. 10).

PND (rec. 5) could imply a possibility to terminate a pregnancy if the foetus has a (likely) pathogenic variant that causes the development of a CPS. Not only the associated risk of miscarriage (estimated as below 0.5% [13]) has to be considered, but also other complications and mostly the severity of the associated CPS. PND is an invasive test and is not recommended if: (1) there is no intention to terminate the pregnancy when a (likely) pathogenic variant is found, (2) one is not able to bear the emotional burden associated with a pregnancy termination, and (3) the local legal regulation does not permit a pregnancy termination.

PGT offers the option to select an embryo which does not carry the familial (likely) pathogenic variant for implantation. PGT-M (for monogenic conditions) avoids the procedure-related risks associated with invasive PND and the psychological burden related to a possible pregnancy termination. However, procedures of in vitro fertilization and embryo transfer can be challenging and may also be a psychological and medical burden. Local legal regulation has to be followed. In some centres it is possible that in a case where all embryos are positive for the (likely) pathogenic variant, the involved individuals may decide to pursue implantation, while in other centres the affected embryos are automatically discarded. This is dependent on local ethical frameworks and legal standing.

Prenatal and preimplantation genetic testing reveal only the presence of a (likely) pathogenic variant and cannot predict individual clinical presentation or prognosis for the majority of CPS. Uncertainty in clinical outcomes is of particular concern for individuals making reproductive decisions in those CPSs with limited or only poorly established genotype-phenotype correlations [14].

Decisions such as testing for a CPS have consequences not only for the individuals but also for other family members and the potential future offspring [15]. Furthermore, it should be noted that gaining knowledge and insight may also cause an increase in psychological burden and challenges in decision making [16–19]. There is no single “right” answer, and all decisions are individual and context dependent. Individuals affected by CPS often state gaps in their knowledge and difficulties in understanding their options, even after receiving reproductive counselling [2]. Therefore, the complexity of reproductive decisions should be acknowledged in counselling.

#### Timing of reproductive counselling provision

The second set of recommendations is 7–11 in Table 2. Reproductive counselling and aiding decision for individuals with a CPS is a dynamic and unpredictable process since patient’s understanding, wishes, desires, and needs change throughout the course of their life [20] (rec. 7, 9, 11). A person’s perception of risk and perspective may also change over time. This could be related to the development of manifestations of the CPS.

There are several key decision points when genetic reproductive counselling is essential (rec. 7) [19, 21–26]: [1] prior to diagnostic testing, [2] at diagnosis of the syndrome, [3] at reproductive age, [4] when actively considering pregnancy, [5] substantial change in clinical course and health condition; incorporating the current family status in the process of counselling is of importance as some options may not be available (rec. 7–11).

Consequently, the reproductive counselling is not a “one and done” intervention, but rather should be considered as a dynamic process at different times as circumstances (clinical and psychological) and options might change (rec. 7, 8).

The ability for an individual to understand potentially complex medical and technical information while also considering the significant challenge of decision making and impact of long-term psychological burden is unique and highly personal. Consideration should therefore be made to break reproductive counselling into multiple sessions over time, by leaving patients room to digest the information, manage their own emotions and to process their thoughts (rec. 7) [14]. Some patients may reject the offer of counselling at one point but change their mind later or vice versa [27] dependent on life circumstances and, especially in CPSs in association to the clinical context (rec.1). It should be made clear that the decision to pursue counselling can be changed at any time without any disadvantage regarding the option of counselling (rec. 4, 7).

#### Presentation of reproductive options

The third set of recommendations 12–14 is given in Table 3. The best practice for reproductive counselling is a multidisciplinary approach. The core disciplines should include [1] clinical geneticist or genetic counsellor, [2] disease-expert who know the clinical spectrum and course of the specific CPS, [3] gynaecologist or/and reproductive specialist (e.g., in case of in vitro fertilization) and [4] psychologist (rec. 6, 12, 13). It is crucial that all available options are explained and offered to the individual with CPS, and, if relevant, to their partner (rec. 2, 3, 14). Centres specializing in certain reproductive options (e.g. in vitro fertilization) can provide specific counselling, but also need to explain other options or refer the patient first to other expert providers for comprehensive counselling. Centres specialized in certain CPSs can collaborate with specialists (geneticist, oncologist, gynaecologist, etc.) to provide a comprehensive counselling. As mentioned in the previous section, multiple sessions and take-away materials can be helpful.

#### Range of assisted reproductive technologies (Table 4)

The fourth set of recommendations 15 and 16 are given in Table 4. Assisted reproductive technologies are a broad term encompassing medical technologies used to support conception and pregnancy, primarily used to address infertility. This subject involves procedures such as in vitro fertilization, intracytoplasmic sperm injection, cryopreservation of gametes and embryos, and the use of donor gametes or embryos. It can also be used outside of an infertility setting in case of preimplantation genetic testing. The need, approval and availabilities of genetic testing in different scenarios of insemination and PND may vary due to national legislation and should be part of the discussion with the patients. All these different options should also be explained to the patient in a counselling (rec. 15, 16).

### Discussion and future direction

Up to now, only one specific guideline on the issue of reproductive counselling has been published: the 2023 British Society of Genomic Medicine guideline [28]. The broad recommendations of the UK and our European guidelines are in concordance. Each of them is adapted to its own local legal regulation and health care systems. While the UK guideline gives specific guidance, for example to the timing of prenatal diagnosis with referral to the UK Abortion act 1967, our guideline focuses more on general timing and strategies to access reproductive counselling, and less on the detailed suggestions on the different reproductive options. This is because our guideline aims to cover all European countries with varying health care systems and possibilities for reproductive interventions. Also, it is important to note that legal and ethical regulation across European countries are diverse, for example, regarding termination of pregnancy.

Some brief recommendations regarding reproductive options are included in several guidelines for specific CPS. For example, recommendation number 6 in the “ERN GENTURIS clinical practice guidelines for the diagnosis, treatment, management and surveillance of people with schwannomatosis” [29], and recommendations 7 and 8 in the “ERN GENTURIS guideline on constitutional mismatch repair deficiency diagnosis, genetic counselling, surveillance, quality of life, and clinical management” [30]. However, such recommendations are more general and abstract, like “patients should be offered reproductive counselling”, which is meant more for general health care professionals and specialists such as oncologists. By contrast, our guideline is meant to assist those who provide the counselling, for example, geneticists and gynaecologists, and it is hoped that the guideline should also encourage patients to request for specific reproductive counselling. This guideline is also meant to initiate the discussion about counselling and diagnostic approaches in individuals with a CPS. As CPSs are mostly complex genetic disorders associated with several challenges, answers cannot be provided by a single diagnostic entity. Moreover, multidisciplinary approaches should be formalised to provide up-to-date genetic counselling and respective diagnostic and therapeutic options.

Reproductive medicine techniques have experienced rapid development [31], and pre-conceptional counselling was rated as the most important pregnancy related issue in a recent survey among clinicians and patient representatives from 20 ERNs [6]. However, the awareness of the applicability of methods such as PGT for CPSs appears to be still limited, among both health professionals and patients [2], and the limited access to specialists in reproductive counselling is a challenge [6].

The formulation of these guidelines for counselling on reproductive options for individuals with a CPS inevitably highlighted areas in which further research is required in order to guide more definitive conclusions. Examples of topics for further research include:

- Centres providing assisted reproductive technology for CPSs should collaborate to pool, analyse and publish data regarding safety and success rates.
- The effectiveness of alternative patient education tools to replace or supplement individualised in-person reproductive counselling should be assessed prior to their introduction into clinical care.
- There would be value in the community around CPSs (both experts and affected individuals) working together to outline an ethical framework regarding the reproductive options and decision-making.
- Condition-specific evidence regarding the ideal form that reproductive counselling should take is lacking.
- The role of lay organizations in the development of clinical care guidelines.
- Development of multinational registers.

## Supplementary information

Supplement
