# Supplementary material for: ERN GENTURIS guideline on counselling on reproductive options for individuals with a cancer predisposition syndrome (including genturis)
Source: Eur J Hum Genet. 2026 Jan 13;34(3):307–13. doi: 10.1038/s41431-025-02007-4 (PMC12963376; doi:10.1038/s41431-025-02007-4)
Supplement: Supplementary file 1 — Supplement [file 41431_2025_2007_MOESM1_ESM.docx]

**Supplement**

Literature search terms:

• ("reproduction"[MeSH Terms] OR "reproduction"[All Fields] OR "reproductions"[All Fields] OR "reproductive"[All Fields] OR "reproductively"[All Fields] OR "reproductives"[All Fields] OR "reproductivity"[All Fields]) AND ("genetic counselling"[All Fields] OR "genetic counseling"[MeSH Terms] OR ("genetic"[All Fields] AND "counseling"[All Fields]) OR "genetic counseling"[All Fields]) AND ("cancer s"[All Fields] OR "cancerated"[All Fields] OR "canceration"[All Fields] OR "cancerization"[All Fields] OR "cancerized"[All Fields] OR "cancerous"[All Fields] OR "neoplasms"[MeSH Terms] OR "neoplasms"[All Fields] OR "cancer"[All Fields] OR "cancers"[All Fields])

Translations

**reproductive**: "reproduction"[MeSH Terms] OR "reproduction"[All Fields] OR "reproductions"[All Fields] OR "reproductive"[All Fields] OR "reproductively"[All Fields] OR "reproductives"[All Fields] OR "reproductivity"[All Fields]

**Genetic counselling**: "genetic counselling"[All Fields] OR "genetic counseling"[MeSH Terms] OR ("genetic"[All Fields] AND "counseling"[All Fields]) OR "genetic counseling"[All Fields]

**cancer**: "cancer's"[All Fields] OR "cancerated"[All Fields] OR "canceration"[All Fields] OR "cancerization"[All Fields] OR "cancerized"[All Fields] OR "cancerous"[All Fields] OR "neoplasms"[MeSH Terms] OR "neoplasms"[All Fields] OR "cancer"[All Fields] OR "cancers"[All Fields]

**829 articles (25th April 2024)**

• (("reproduction"[MeSH Terms] OR "reproduction"[All Fields] OR "reproductions"[All Fields] OR "reproductive"[All Fields] OR "reproductively"[All Fields] OR "reproductives"[All Fields] OR "reproductivity"[All Fields]) AND ("genetic counselling"[All Fields] OR "genetic counseling"[MeSH Terms] OR ("genetic"[All Fields] AND "counseling"[All Fields]) OR "genetic counseling"[All Fields]) AND ("Schwannomatosis"[All Fields] OR "neurofibromatosis"[All Fields] OR "Adenomatous polyposis syndrome"[All Fields] OR "Hamartomatous polyposis syndrome"[All Fields] OR "Lynch syndrome"[All Fields] OR "Hereditary Non-Polyposis Colorectal Cancer"[All Fields] OR "Hereditary Breast and Ovarian Cancer syndrome"[All Fields] OR "PTEN"[All Fields] OR "Li Fraumeni syndrome"[All Fields] OR "Birt-Hogg-Dube Syndrome"[All Fields] OR "BRCA1"[All Fields] OR "BRCA2"[All Fields] OR "Familial Malignant Melanoma"[All Fields] OR "Constitutional Mismatch Repair Deficiency"[All Fields] OR "Carney Complex"[All Fields] OR "Hereditary Papillary Renal Cell Carcinoma"[All Fields] OR "Ataxia-Telangiectasia"[All Fields] OR "Bloom syndrome"[All Fields] OR "Gorlin syndrome"[All Fields] OR "Nevoid basal cell carcinoma syndrome"[All Fields] OR "Werner Syndrome"[All Fields] OR "Hereditary Leiomyomatosis"[All Fields] OR "von Hippel-Lindau disease"[All Fields] OR "Fanconi anemia"[All Fields] OR "Hereditary pheochromocytoma-paraganglioma"[All Fields] OR "Paraganglioma"[All Fields] OR "Heritable TP53-related cancer syndrome"[All Fields])

**220 articles (20th May 2024)**

Supplemental Table 1: The ERN GENTURIS guideline group for counselling on reproductive options for individuals with a cancer predisposing syndrome (CPS) including genetic tumour risk syndrome (genturis; ERN GENTURIS counselling on reproductive options guideline group). The members, their expertise/role and affiliation are given in alphabetical order. The members of the core working group (n=6) are listed first and indicated with a green background.

| Author | Speciality/Role | Affiliation |
| --- | --- | --- |
| Said C. Farschtschi, MD | Physician, registrar for neurology and genetic counselling | University Medical Center Hamburg-Eppendorf, Hamburg, Germany  Member of ERN GENTURIS |
| Sandra Janssens, MD, PhD | Clinical geneticist | University Hospital Ghent, Ghent, Belgium  Member of ERN GENTURIS |
| Candy Kumps, MD, PhD | Clinical geneticist | University Hospital Ghent, Ghent, Belgium  Member of ERN GENTURIS |
| Tamara Hussong Milagre | Community representative | EVITA Association – Hereditary Cancer (Associação EVITA – Cancro Hereditário, Portugal  ePAG of ERN GENTURIS |
| Sarah Pugh | Consultant Genetic counsellor | Manchester Centre for Genomic Medicine, Manchester University Foundation NHS trust, Manchester, United Kingdom |
| Laura Kirstine Sønderberg Roos, MD, PhD | Clinical geneticist (chair) | Rigshospitalet, Copenhagen, Denmark  Member of ERN GENTURIS |
| Anna Sophie Berghoff, MD, PhD | Oncologist | Medical University of Vienna, Vienna, Austria  Member of ERN GENTURIS |
| Estela Carrasco López, MSc | Genetic counsellor | Vall d'Hebron University Hospital, Barcelona, Spain  Member of ERN GENTURIS |
| Claudia Cesaretti, MD | Clinical geneticist | Fondazione IRCCS Ca' Granda, Ospedale Maggiore Policlinico, Milan, Italy Member of ERN GENTURIS |
| Ellen Denayer, MD, PhD | Clinical geneticist | University Hospitals Leuven, University of Leuven, Leuven, Belgium Member of ERN GENTURIS |
| Francesca Fianchi, MD | Internist | Fondazione Policlinico Universitario A. Gemelli IRCCS, Rome, Italy  Member of ERN GENTURIS |
| Marianne Geilswijk, MD | Clinical geneticist | Aarhus University Hospital, Aarhus, Denmark  Member of ERN GENTURIS |
| Mariëtte van Kouwen, MD, PhD | Gastroenterologist | Radboud university medical center, Nijmegen, the Netherlands  Member of ERN GENTURIS |
| Periklis Makrythanasis, MD, PhD | Medical geneticist | 'Aghia Sophia' Children's Hospital, National and Kapodistrian University of Athens, Athens, Greece  Member of ERN GENTURIS  University of Geneva, Geneva, Switzerland  Biomedical Research Foundation of the Academy of Athens, Athens, Greece |
| Renata d' Oliveira | Clinical geneticist | Unidade Local de Saúde (ULS) São João, Porto, Portugal  Member of ERN GENTURIS |
| Claas Röhl | Community representative | NF Kinder/ NF Patients United, Vienna, Austria  ePAG of ERN GENTURIS |
| Diana Salinas-Chaparro, MSc | Genetic counsellor | Hospital Sant Joan de Deu, Barcelona, Spain  Member of ERN GENTURIS |
| Ileen Slegers, MSc | Genetic counsellor, midwife specialist | UZ Brussel, Brussel, Belgium  Member of ERN GENTURIS |
| Irene Spinelli, MD | Gastroenterologist | Fondazione Policlinico Universitario A. Gemelli IRCCS, Rome, Italy  Member of ERN GENTURIS |
| Manon Suerink, MD, PhD | Clinical geneticist | Leiden University Medical Center, Leiden, the Netherlands  Member of ERN GENTURIS |
| Salvo Testa | Community representative | President Fondazione Mutagens (hereditary syndromes carriers), Milano, Italy  ePAG of ERN GENTURIS |
| Ariane Van Tongerloo | Psychologist | University Hospital Ghent, Ghent, Belgium  Member of ERN GENTURIS |
| Eva Trevisson, MD, PhD | Clinical geneticist | University of Padua, University Hospital of Padua, Padua, Italy  Member of ERN GENTURIS |

Supplemental Table 2: Additional experts that participated in the Delphi survey, their expertise/role and affiliation in alphabetical order.

| Name | Speciality / Role | Healthcare provider |
| --- | --- | --- |
| Amedeo Azizi, MD | paediatrician | Medical University of Vienna, Vienna, Austria  Member of ERN GENTURIS |
| Manuela Bapista, MD | surgeon | ULS São João, Porto, Portugal  Member of ERN GENTURIS |
| Tanya M. Bisseling, MD, PhD | gastroenterologist | Radboud university medical center, Nijmegen the Netherlands  Member of ERN GENTURIS |
| Ignacio Blanco, MD, PhD | clinical geneticist and surgeon | Hospital germans Trias i Pujol, Barcelona, Spain  Member of ERN GENTURIS |
| Alejandra Cano, MSc | psycho-oncologist | Hospital Universitario Vall d'Hebron, Barcelona, Spain  Member of ERN GENTURIS |
| Melchor Carbonell Socias, PhD | gynaecologist | Hospital Universitario Vall d'Hebron, Barcelona, Spain  Member of ERN GENTURIS |
| Andrada Ciuca, PhD | clinical psychologist and genetic counsellor | Babes-Bolyai University, Cluj, Romania |
| Lotte Berdiin Colmorn, MD, PhD | consultant / genetic counsellor | Rigshospitalet, Copenhagen, Denmark  Member of ERN GENTURIS |
| Samantha Doyle, MD | reproductive geneticists | The National Maternity Hospital, Dublin, Ireland |
| Francesca Filippi, MD | gynaecologist | Fondazione IRCCS Ca' Granda, Ospedale Maggiore Policlinico, Milan, Italy Member of ERN GENTURIS |
| Lenka Foretova, MD, PhD | clinical geneticist | Masaryk Memorial Cancer Institute, Brno, Czech Republic  Member of ERN GENTURIS |
| Sophie Frank, MD | gynaecologist | Institut Curie, Paris, France  Member of ERN GENTURIS |
| Jacek      Gronwald, MD, PhD | geneticist | Pomeranian Medical University - University Clinical Hospital no 1, Szczecin, Poland  Member of ERN GENTURIS |
| Hildegunn Høberg Vetti, MD, PhD | geneticist | Haukeland University Hospital, Bergen, Norway  Member of ERN GENTURIS |
| Marek Karwacki, MD, PhD | paediatrician | Medical University of Warsaw University Clinical Center, Warsaw, Poland |
| Antonis Kattamis, MD | paediatric haematologist, oncologist | Aghia Sophia Children's Hospital, National and Kapodistrian University of Athens, Athens, Greece  Member of ERN GENTURIS |
| Mateja Krajc, MD, PhD | geneticist | Institute of Oncology Ljubljana, Ljubljana, Slovenia  Member of ERN GENTURIS |
| Barbara Laga | psychologist | University Hospital Gasthuisberg, Leuven, Belgium  Member of ERN GENTURIS |
| Marie Louise Landsvig | trained midwife, genetic counsellor | Rigshospitalet, Copenhagen, Denmark  Member of ERN GENTURIS |
| Milan Macek, MD | geneticist | Charles University and Motol University Hospital, Prague, Czechia  Member of ERN GENTURIS |
| Dolors Manau, MD, PhD | gynaecologist | Hospital CLíNIC of Barcelona, Barcelona, Spain |
| Angela Mastronuzzi, MD, PhD | paediatric oncologist | IRCCS Bambino Gesù Children's Hospital, Rome, Italy  Member of ERN GENTURIS |
| Lorena Moreno Calle, MSc | Genetic Counsellor | Hospital CLíNIC of Barcelona, Barcelona, Spain |
| Kai Muru, MD, PhD | clinical geneticist | Tartu University Hospital, University of Tartu, Tartu, Estonia  Member of ERN GENTURIS |
| Federica Natacci, MD | Clinical geneticist | Fondazione IRCCS Ca' Granda, Ospedale Maggiore Policlinico, Milan, Italy Member of ERN GENTURIS |
| Valeria Nicotra, MD | Clinical geneticist | Fondazione IRCCS Ca' Granda, Ospedale Maggiore Policlinico, Milan, Italy Member of ERN GENTURIS |
| Rosie O Shea, PhD | Genetic counsellor | Trinity College Dublin, St. James’s Hospital, Dublin, Ireland  School of Medicine and Health, University of Sydney, Australia |
| Minna Pöyhönen, MD, PhD | geneticist | Helsinki University Hospital, Helsinki, Finland  Member of ERN GENTURIS |
| Tiia Reimand, MD, PhD | medical geneticist | Tartu University Hospital, Tartu, Estonia  Member of ERN GENTURIS |
| Alessandra Renieri | geneticist | AOU Senese, Siena, Italy  Member of ERN GENTURIS |
| Kerstin Rhiem, MD, PhD | gynaecologist | Center for Familial Breast and Ovarian Cancer, Center for Integrated Oncology (CIO), University Hospital Cologne, Cologne, Germany |
| María Constanza Roa Bravo | geneticist in training | Institut für Klinische Genetik, Universitätsklinikum Carl Gustav Carus, Dresden, Germany  Member of ERN GENTURIS |
| Kleoniki Roka, MD, PhD | paediatrician | “Aghia Sophia” Children’s Hospital, Athens, Greece  Member of ERN GENTURIS |
| Andrea Ros, MSc | genetic counsellor | Germans Trias i Pujol Hospital, Barcelona, Spain  Member of ERN GENTURIS |
| Susanne Schüler-Toprak, MD | gynaecologist | University Medical Center Regensburg, Regensburg, Germany |
| Fabiana Sousa, MD | surgeon | Unidade Local de Saúde (ULS) São João, Porto, Portugal  Member of ERN GENTURIS |
| Verena Steinke-Lange, MD | geneticist | MGZ - Medical Genetics Center, Munich, Germany  Member of ERN GENTURIS |
| Isobel Turbin | Principal Genetic Counsellor | Cambridge University Hospitals NHS Foundation Trust, Cambridge, UK |
| Alexander E. Volk, MD | clinical geneticist | Institute of Human Genetics, University Medical Center Hamburg-Eppendorf, Germany  Member of ERN GENTURIS |
| Karin A.W. Wadt, MD, PhD | clinical geneticists | University of Copenhagen, Copenhagen, Denmark  Rigshospitalet, Copenhagen, Denmark  Member of ERN GENTURIS |
| anonymous | gyn oncologist | Italy |

Supplemental Table 3: General considerations

In the following, the term cancer predisposition syndrome (CPS) will be used. Therefore, the term “individual with a CPS” will refer to individuals that are diagnosed with a CPS, including all individuals with a genturis. The value and need for reproductive counselling are relevant for all individuals with CPS, thus also including genturis patients.

| With respect to the hereafter formulated recommendations, the following^*^ should be taken into account: |
| --- |
| **Healthcare professionals should always**:   - respect the individual’s autonomy and personal readiness while ensuring access to necessary information. - provide information in a timely manner, tailored to the individual’s needs and circumstances considering that what is timely may vary based on personal and healthcare system factors. - provide up-to-date information, recognising that clinical screening strategies, treatment guidelines, diagnostic criteria, nomenclature, reproductive methods, and genetic techniques may change at short notice as scientific knowledge evolves. |
| **Healthcare centres providing counselling on reproductive options for individuals with a cancer predisposition syndrome and relevant family members should:**   - counsel patients prospectively in advance about PND and preimplantation genetic testing, including its medical procedure, limitations, psychological impact, success rates, and the possibility of obtaining only affected embryos/foetuses. - clearly present and explain all available reproductive options to patients, including those beyond prenatal diagnosis and preimplantation genetic testing (such as sperm/oocyte donation, adoption, and postnatal diagnosis). This should include information on reproductive window, waiting times and delays (such as the time required to obtain test results for prenatal diagnosis or the timeline to the first embryo transfer in preimplantation genetic testing) - ensure realistic expectations and informed decision-making, tailored to the patient’s reproductive potential, before initiating any procedures. For example, a 38-year-old woman may have lower success rates in PGT-M procedures due to her ovarian reserve and oocyte quality compared to a 28-year-old woman. - Provide guidance in accordance with country-specific legal possibilities and processes. For example, some countries may require ethical board approval for PND or PGT in individuals with a cancer predisposition syndrome on an individual basis, and not all cases may be accepted. - Include information about the availability of public funding for PGT, if applicable. - Facilitate liaison with IVF clinics regarding fertility potential, including consideration of the patient’s age and ovarian reserve (e.g., AMH levels), to set realistic expectations for success rates. |
| **Reproductive counselling**  Personal philosophies, religion, cultural values, and individual preferences concerning family and reproduction significantly influence attitudes towards prenatal diagnosis and preimplantation genetic testing. Counsellors should be sensitive to and understand these perspectives, ensuring non-judgemental, personalised, and non-directive support. |
| ^*^ these general statements are based on recommendations included in the first Delphi round which passed the threshold for consensus. |
